# Accumulation of trace element content in the lungs of Sao Paulo city residents and its correlation to lifetime exposure to air pollution

**DOI:** 10.1038/s41598-022-15048-2

**Published:** 2022-06-30

**Authors:** Nathália Villa dos Santos, Carolina Leticia Zilli Vieira, Paulo Hilario Nascimento Saldiva, Carmen Diva Saldiva De André, Barbara Paci Mazzilli, Maria de Fátima Andrade, Catia Heloisa Saueia, Mitiko Saiki, Mariana Matera Veras, Petros Koutrakis

**Affiliations:** 1 Laboratory of Environmental and Experimental Pathology LIM05, Department of Pathology, University of Sao Paulo School of Medicine, São Paulo, SP Brazil; 2 Department of Environmental Health, Harvard T.H. Chan School of Public Health, Boston, MA USA; 3 Nuclear and Energy Research Institute, IPEN-CNEN, São Paulo, SP Brazil; 4 Institute of Mathematics and Statistics, University of Sao Paulo, Sao Paulo, Brazil; 5 Department of Environmental Health School of Public Health, University of São Paulo, Brazil, Av. Dr. Arnaldo, 715, São Paulo, Brazil; 6 Atmospheric Sciences Department, Institute of Astronomy, Geophysics and Atmospheric Sciences, University of São Paulo, São Paulo, Brazil

**Keywords:** Chemical biology, Environmental sciences, Diseases

## Abstract

Heavy metals are natural and essential elements of the environment and living beings, produced from natural (e.g. volcanic activity and cosmic ray-induced spallation) and anthropogenic processes (e.g. industrial and fossil fuel combustion). High-concentrations of heavy metals and radionuclides are also originated from anthropogenic activities in urban and industrial areas. In this preliminary study, we analyzed the levels of heavy metals and Polonium-210 (^210^Po) in lung tissues in autopsies from residents of the city of Sao Paulo, SP, Brazil. In order to identify the link among sources of the heavy metals in lungs, factor analysis was performed. Of the first four factors, which explain 66% of the total variability, three were associated with vehicular sources. The fitting of a regression model with ^210^Po as the response variable and with the four factors as explanatory variables, controlling for age, sex and tobacco, showed a significant association between the concentration of polonium and the first factor that is generated by catalysts and brakes (coefficient = 0.90, standard error = 0.33, p = 0.016). Our findings suggest an association between traffic-related trace metals and ^210^Po in lung autopsies.

## Introduction

Degradation and contamination of the environment (air, water and soil) by toxic substances is a common problem in urban areas, caused mainly by human activities (industries, vehicular emissions, unregulated disposal of solid waste)^1^. Among these contaminants, some metals deserve to be highlighted because they are toxic for living beings even at low concentrations. These toxic metals, some commonly known as heavy metals, occur naturally in soil at low concentrations^2^. However, studies have detected high concentrations of heavy metals in water for human consumption^3^. They are also associated with urban and industrial pollution especially in particulate matter suspended in the air^4^.

Exposure to heavy metals can occur upon inhalation of contaminated air, by ingestion of contaminated water or food, by contact with contaminated soil/dust, and by use of cosmetic products. In Brazil there are laws to limit metals in food (Arsenic, Lead, Cadmium, Mercury and Tin)^5^, in water (for example, arsenic, cadmium, chromium, copper, lead, mercury, nickel, silver, zinc), and in air pollution (Lead)^4,6^. However, there is no specific program of monitoring the presence and levels of these metals in the different environments^7^, except presence of lead in the air.

In general, health effects of these toxic metals depend on route, dose and duration of the exposure, as well as the age of the exposed individual^8^. For example, cadmium, lead and mercury have been linked to damage in kidney, bone and lungs, and neurobehavioral and developmental disorders in fetuses, infants and children^1^. Heavy metals are bioaccumulative in ecosystems, and can be biomagnified in animals consumed by humans^1^. Moreover, long-range transport of heavy metals in air pollution to remote places far from the sources of emission has been recognized as an important factor when assessing contamination of the environment and health effects in humans^31^.

Heavy metals are commonly attached to air particulates (PM), especially those in the submicron category (0.1–1 µm)^4^. Fortoul et al. (1996) showed a significant increase of heavy metal concentrations in lung autopsies linked to urban and industrial air pollution in residents of the City of Mexico from 1950 to 1980’s^9^. Our previous study showed that Polonium-210 (^210^Po) levels in autopsies were linked to urban pollution and tobacco smoke^10^. ^210^Po decay onto its intermediate heavy metal progenies Bismuth-210 (^210^Bi) and lead-210 (^210^Pb) after 138 days in the environment^11^, and these metals may deposit and accumulate for years in lungs.

PM is a complex mixture of solid and liquid particles suspended in air, whose physicochemical properties and biological effects vary with location and time^12^. PM plays a major role in transport of heavy metal radioactivity^13^. Regional differences in PM mortality risk are frequently attributed to geographic variability in PM composition, spatial heterogeneity of constituents, topography, emission/removal rate, transport and dispersion, and differences in population risk^4,14,15^. A recent study showed high concentrations of heavy metal in tree rings in the city of Sao Paulo (Brazil)^11^ and their associations with air pollution, reinforcing the importance of vehicular emissions to the degradation of air quality and health risks^16,17^.

Therefore, in this study our objective was to investigate the association between the concentrations of heavy metal trace elements and ^210^Po in lung autopsies performed in the Post Mortem Verification Service of the City of São Paulo (SVOC) and lifetime exposure to air pollution in Sao Paulo city, Brazil.

## Methods

### Tissue samples

This study was approved by the Research Ethics Committee of the University of Sao Paulo (number 0.002.065) and inclusion criteria were: age equal or greater than 18 years, living in the Metropolitan Area of Sao Paulo (MASP) for at least 3 months, have one close relative to provide reliable and complete information during the interview to retrieve information about other sources of exposure, and not presenting macroscopic alterations of the lungs, brain orthe olfactory epithelium. Fresh samples of lungs were obtained from 20 individuals during autopsy procedures at the SVOC (Death Verification Service of the Capital).

In brief, right after the body was claimed by a relative (next-of-kin “NOK”) who had signed to allow the autopsy procedure, a trained interviewer invited the NOK to participate in our study by explaining its purpose and relevance. Upon acceptance, the relative was taken to a private room where an informed consent form was voluntarily signed, authorizing the collection of lung images and tissue samples. Then, a questionnaire about previous health conditions, residential address, sociodemographic details, life habits, smoking status (smoker or former smoker, or “environmental tobacco smoke”, which includes non-smoking individuals who lived with smokers), occupation, time of residence in the MASP, and time spent commuting was completed (see supplemental materials).

### Anthracosis index

The anthracosis index was measured *in* the pleural surface of the lungs as described previously by Takano et al.^18^.

### Lung sample preparation

Lung tissue samples from upper lobe (20–37 g) were placed separately in clean polyethylene bags and kept at −80 ^o^C prior to transport to the Nuclear and Energy Research Institute (IPEN-CNEN/SP). Special care was taken during handling to avoid external contamination by metals, using plastic tools and powder-free surgical gloves. The tissues were rinsed using purified water in order to remove the blood. The tissues were homogenized using a titanium knife. Samples were freeze-dried until their constant weight was obtained. In this process of freeze-drying mean weight, losses (in percentage) were obtained: 82.0 ± 6.0 for lung tissues. The dried samples were ground manually to obtain a fine powder and placed in polyethylene vials. Prepared samples were stored in a refrigerator at −20 °C prior to neutron activation analysis (NAA).

### Determination of ^210^Po in human samples

Determination of ^210^Po in the lung tissue samples was conducted according to the methods described by Villa et al.^19^.

### Neutron activation analysis procedure

Aliquots of about 150 mg of dried tissue samples were irradiated in the IEA-R1 nuclear reactor along with the synthetic standards of the elements^20^. Short and long period irradiations with a thermal neutron flux of about 4 × 10 12 n cm^–2^ s^–1^ were performed for determination of elements, including Br, Ca, Ce, Cl, Cr, Co, Cs, Fe, Hf, K, La, Mn, Na, Rb, Sb, Sc, Se. Th and Zn. After adequate decay times, the irradiated samples and standards were measured using a Model GC3019 hyperpure Ge detector coupled to a Digital Spectrum Analyzer DSA 1000, both from Canberra. Spectra were collected and processed using Canberra Genie 2000 Version.

Samples and standards were measured at least twice for different decay times. Counting times from 200 to 50,000 s were used, depending on the half- lives or activities of the radioisotopes considered. The radioisotopes measured were identified according to their half-lives and gamma ray energies. The element concentrations were calculated by the comparative method.

### Statistical analysis

In order to identify the sources of the element traces in the lung tissues, a factor analysis was performed^21^. The applied method was the Principal Components based on a robust correlation matrix composed of Spearman's correlation coefficients between element concentrations, with Varimax rotation.

The association between ^210^Po and the factors representing the metals, adjusted for age, sex, and tobacco use, was initially assessed using multiple linear regression fitted by least squares. A residual analysis suggested the existence of outliers and robust regression models based on MM-estimators were considered. Non-significant variables and interactions were dropped from the initial model step by step of the fitting process. The models were fitted via the *rlm* function from the MASS library in the R software package^22^.

### Ethics statement

This study is part of the MetroHealth subproject of a project entitled The Use of Modern Autopsy Techniques to Investigate Human Diseases (MODAU) and was approved by the Research Ethics Committee of the University of Sao Paulo (number 2013/21728).

### Human experiment statement

All legal guardians signed the informed consent form before the pathological procedures (supplementary material). This study was approved by the Research Ethics Committee of the University of Sao Paulo (number 2013/21728) in accordance with relevant regulations.

## Results

Descriptive statistics for individual characteristics, and ^210^Po in the lungs and pleural anthracosis are presented in Table 1. The standard deviation of ^210^Po is greater than its mean, suggesting an asymmetrical distribution of actual positive values. This result is illustrated in the box-plot presented in the Figure S1 in the supplementary material.

**Table 1:** Descriptive statistics of the individual characteristics, ^210^Po-Lung and anthracosis.

Variable	N	Mean	StDev (*)	Minimum	Median	Maximum	IQR (**)
**Quantitative variables**							
Age (Years)	20	69.0	18.3	28	71	93	31.8
Years living in Sao Paulo	20	50.7	13.0	21	50	69	15.8
Daily commuting (h)	19	1.7	2.2	0.0	0.7	8.0	3.0
Anthracosis	16	0.22	0.15	0.00	0.19	0.55	0.23
^210^Po	20	2.66	3.08	0.37	1.48	12.61	2.05
**Qualitative variables**							
Male	N = 12	(57.1%)					
Smoker or former smoker	N = 8	(38.1%)					
Environmental tobacco smoke	N = 11	(36.6%)					

(*) standard deviation; (**) interquartile range.

The observed concentrations of Br, Ca, Ce, Cl, Cr, Co, Cs, Fe, Hf, K, La, Mn, Na, Rb, Sb, Sc, Se, Th and Zn are summarized in Table 2, and their distributions are represented in the box-plots in Figure S2. These results indicate high variability of the elements concentrations. The Spearman correlation coefficients between the concentrations of the elements are shown in Table S1.

**Table 2:** Descriptive statistics of the concentrations of chemical elements in the lung (µg/kg).

Metal	N	Mean	StDev (*)	Minimum	Median	Maximum	IQR(**)
Br	20	40.4	55.5	15.3	26.4	273.6	15.1
Ca	20	2756.0	6310.0	564.0	1080.0	29,319.0	992.0
Ce	19	294.5	235.0	39.6	245.6	1019.4	261.9
Cl	20	16,229.0	4995.0	10,690.0	15,901.0	27,401.0	6314.0
Co	20	93.7	34.8	25.7	94.0	149.0	60.4
Cr	20	1047.0	906.0	121.0	695.0	3743.0	1111.0
Cs	20	141.8	51.1	20.4	144.1	211.9	80.8
Fe	20	1090.9	428.1	398.4	980.3	1784.5	731.3
Hf	18	22.8	21.4	4.6	16.8	77.1	20.5
K	20	7558.0	1324.0	5289.0	7158.0	9663.0	2343.0
La	20	222.4	124.5	61.4	213.1	465.2	185.8
Mn	20	702.4	313.9	182.9	657.0	1669.0	330.2
Na	20	10,339.0	3466.0	6345.0	10,308.0	20,858.0	4154.0
Rb	20	16.7	3.8	11.1	16.3	23.9	6.9
Sb	20	193.4	139.9	16.6	164.8	591.4	135.2
Sc	20	29.1	30.9	3.6	22.5	124.0	22.0
Se	20	499.8	137.8	228.0	472.5	871.0	180.0
Th	20	37.5	32.0	2.7	30.8	113.3	33.3
Zn	20	55.8	11.5	42.5	52.2	87.4	15.1

(*) standard deviation; (**) interquartile range.

Table 3 contains the rotated factor loadings of the heavy metals in the 6 first factors. The number of factors was selected based in the scree plot in Figure S3. Together, these six factors account for 84% of the total data variability. Based on these results the factors can be interpreted as follows: Factor 1 is associated with the catalyst material and the vehicle brakes, Factor 2 is related to metals in the lung tissue itself. Factors 3 and 4 represent metals related to vehicular emissions. Factor 5 don’t have interpretation and factor 6 is related to soil resuspension^23,24^. The communalities are high, and thus the sample size has less impact on the accuracy of the estimates^25^.

**Table 3:** Rotated factor loadings. Values greater than 0.5 are highlighted in bold. The percentage of total variance explained by each factor is also presented in the table.

Metal	Factor1		Factor2	Factor3	Factor4	Factor5	Factor6	Communality
Br	0.44		0.11	**0.50**	**−0.58**	**−**0.01	**−**0.14	0.81
Ca	**−**0.20		**−**0.35	**−**0.42	**−0.54**	**−**0.12	0.27	0.73
Ce	0.45		0.58	**−**0.01	**−**0.30	**−**0.28	0.40	0.87
Cl	**−**0.43		**−**0.33	**0.68**	**−**0.25	0.12	0.21	0.87
Co	**0.81**		**−**0.23	**−**0.13	0.20	**−**0.42	0.02	0.94
Cr	**0.67**		**−**0.18	0.07	0.51	0.05	0.36	0.87
Cs	**0.54**		0.21	**−**0.40	**−**0.04	0.53	0.12	0.79
Fe	0.33		0.20	0.26	0.11	**−**0.39	**−**0.66	0.81
Hf	**0.88**		**−**0.06	**−**0.05	0.07	0.13	**−**0.11	0.81
K	**−**0.32		**0.59**	**−**0.12	0.04	**−**0.55	0.14	0.78
La	**0.61**		0.49	0.30	**−**0.25	0.16	0.27	0.85
Mn	**0.63**		**−**0.07	0.19	**−**0.22	0.34	**−**0.43	0.79
Na	**−**0.69		**−**0.36	0.42	**−**0.04	**−**0.03	0.10	0.79
Rb	**−**0.40		**0.65**	**−**0.21	0.25	0.46	**−**0.12	0.91
Sb	**0.71**		**−**0.54	0.11	0.01	0.18	0.19	0.87
Sc	**0.95**		0.02	**−**0.06	0.02	0.01	0.02	0.90
Se	**−**0.22		−0.68	−0.42	0.05	−0.06	−0.13	0.71
Th	**0.92**		−0.19	0.06	0.07	−0.30	0.04	0.98
Zn	0.15		−0.08	−**0.57**	−**0.68**	−0.06	−0.20	0.86
%Variance explained	0.35		0.14	0.11	0.09	0.08	0.07	0.84

The first four interpretable factors in addition to age, sex and tobacco, were considered as explanatory variables in an initial regression model, with ^210^Po concentration as the response variable. In a backward robust model fitting process, predictor variables that had no additional significant contribution to explain the concentration ^210^Po, in the presence of the other predictors, were excluded from the model, one by one. A summary of the results in the final step of the model fitting is presented in Table 4. The results indicate that, for individuals of the same age and sex, ^210^Po concentration tends to increase with the increase in vehicle emissions.

**Table 4:** Results in the final regression model with ^210^Po as response variable.

Predictor	Coefficient	Standard error	t-value
Intercept	6.7	1.4	4.64
Male	−1.9	0.50	−3.88
Age (years)	−0.05	0.02	−2.86
Factor 1	0.90	0.33	2.73

## Discussion

Measurements of the concentrations of trace metals and ^210^Po in human lung tissue from São Paulo city residents indicated that there is a close association between exposures to air pollution. The urbanization process has promoted a better quality of life, however, it has also caused a series of environmental impacts that directly or indirectly affect human health, such as air pollution. In Sao Paulo city, weak national and local environmental legislation, unplanned urbanization, industrial structure, unsustainable pattern of energy consumption and generation, and road-based transportation are major drivers of our deteriorated air quality.

The effects of air pollution on human health vary from moderate to severe and depend on the concentrations to which individuals are exposed, their composition, age, and the presence of preexisting diseases. The presence of heavy metals associated with fine PM is one of the determinants of health risks posed by air pollution. Different studies that evaluated the elemental composition of PM from Sao Paulo city have shown positive correlations for the following parameters: SO4(2–), NO3–, NH4+, Zn, Fe, Al, Ba, Cu, Pb, Mn and Ni^26^. Heavy metals in the atmosphere are attached to PM, with sources that include metal-enriched sewage, mining and industrial activities, and automotive emissions^37^. PM is composed largely of organic and elemental carbon,, including incomplete combustion of biomass materials fossil fuels^37^. Other sources that are unrelated to the combustion process include brake, tire, clutch, and road surface release^27–30^. The toxics health effects of heavy metals is related to the generation of reactive oxygen and nitrogen species. The toxicity of these metals is primarily due to imbalance between pro-oxidant and antioxidant homeostasis. Indeed, these metals have high affinity for enzymes and proteins containing thiol groups, which are responsible for normal cellular defense mechanisms, and long-term exposure to these metals could lead to apoptosis due to binding of heavy metals to DNA and nuclear proteins^31^. In this study we demonstrated that the presence of Po210 and heavy metals in lungs of Sao Paulo city residents is associated with vehicular emission/air pollution. Other metals detected in lung tissues were also related to exposure to air pollution, time of residence in the city, and time spent commuting.

After inhalation, Radon and its progeny can deposit somewhere along the respiratory tract, increasing the risk of cancer^2^ and non-cancer diseases^32^. Radon is the second leading cause of lung cancer, resulting in approximately 21,000 lung cancer deaths each year in the US (USEPA). In Europe, it is estimated that approximately 3,000 deaths are caused by exposure to radon annually. The World Health Organization (WHO) estimates that radon is the leading cause of lung cancer, accounting for up to 14% of cases worldwide^13^. Rn decay chain produces a series of short- and long-lived alpha and beta particle emitting progeny, including polonium-210 (^210^Po) and lead-210 (^210^Pb), respectively. Alpha particles emitted by these progeny cause more damage since they penetrate only a short distance into tissues, while beta particles from the same progeny penetrate much deeper and cause less damage^33^.

Some metals are required for different cellular functions and homeostasis, however, depending on the dose and exposure duration they can have toxic affects on different physiological processes. Most of the evidence for the negative effects of inhaling heavy metals inhalation come from occupational exposures^34^. For example, occupational exposure to cobalt metal or cobalt-containing hard metal is associated with respiratory effects such as asthma, interstitial lung disease, wheezing, and dyspnea^35^. Studies on the effects of environmental exposure to low levels of heavy metals in humans, such as chromium, are scarce, however prolonged occupational exposures are related to negative effects on the respiratory system, kidney, liver, and cancer^36^. Heavy metals are bioaccumulative in ecosystems, and can be biomagnified in animals that are consumed by humans^1^. Long-range transport of heavy metals in air pollution to places remote from the sources of emission has been recognized as an important factor affecting the environment and human health^39^. Heavy metals are commonly attached to air particulates (PM), especially those in the submicron category (0.1–1 µm)^4^. Fortoul et al. (1996) showed high concentrations of heavy metals linked to urban and industrial air PM in lung autopsies of residents of the Mexico City from the 1950 to 1980’s^40^.

Zinc is found as ZnO in particulate air pollution^41^, and depending on the inhaled concentration it can induce respiratory distress in response to metal deposition^42^.

A meta-analysis conducted by Catalini et al.^43^ evaluated the concentrations of metals in lung tissue as factors involved with the development of lung cancer. Results have shown that Zn and Cu were the metals more represented, and there is no clear relationship between concentration and age. Just one study included in the meta-analysis found a positive correlation between Ni and Cr concentrations and age^44^. Among smokers, concentrations of Al, Cd, Cr, Ni, Pb and Mn were higher, but results could be biased by past occupational exposure to metals^45^. Cr and Pb showed the highest concentrations.

Although this study is only descriptive, using a small sample, it has many strengths. The detailed interview with a family member about the individual's daily activities, potential occupational exposure, tobacco use, time spent in traffic, and the assessment of lifetime exposure using the pulmonary anthracosis index, made it possible to control for factors that could bias the results of the analysis. The analysis and characterization of the presence of these metals in the lungs of residents of the city of Sao Paulo allows us to more plausibly infer and suggest the contribution of the exposure to air pollution to different diseases associated with exposure to heavy metals and radioactive elements, such as lung cancer, hematological and neurodegenerative diseases.

This preliminary study has limitations, including the relatively small number of subjects and the lack of quantification of personal exposures to environmental radon and other air pollutants. A study with a larger population could possibly show significant associations between air pollution and personal characteristics, such as gender, age, and occupation.

In addition, understanding the presence of metals in human tissues may allow us to identify and reduce new sources of exposure and create measures to protect human health.

## Data availability statement

The datasets generated during and/or analyzed during the current study are available from the corresponding author on reasonable request.

## Supplementary Information

Supplementary Information 1.

Supplementary Information 2.

Supplementary Information 3.

Supplementary Information 4.
