# Supplementary figures and images for: Accumulation of trace element content in the lungs of Sao Paulo city residents and its correlation to lifetime exposure to air pollution
Source: Sci Rep. 2022 Jun 30;12:11083. doi: 10.1038/s41598-022-15048-2 (PMC9247064; doi:10.1038/s41598-022-15048-2)

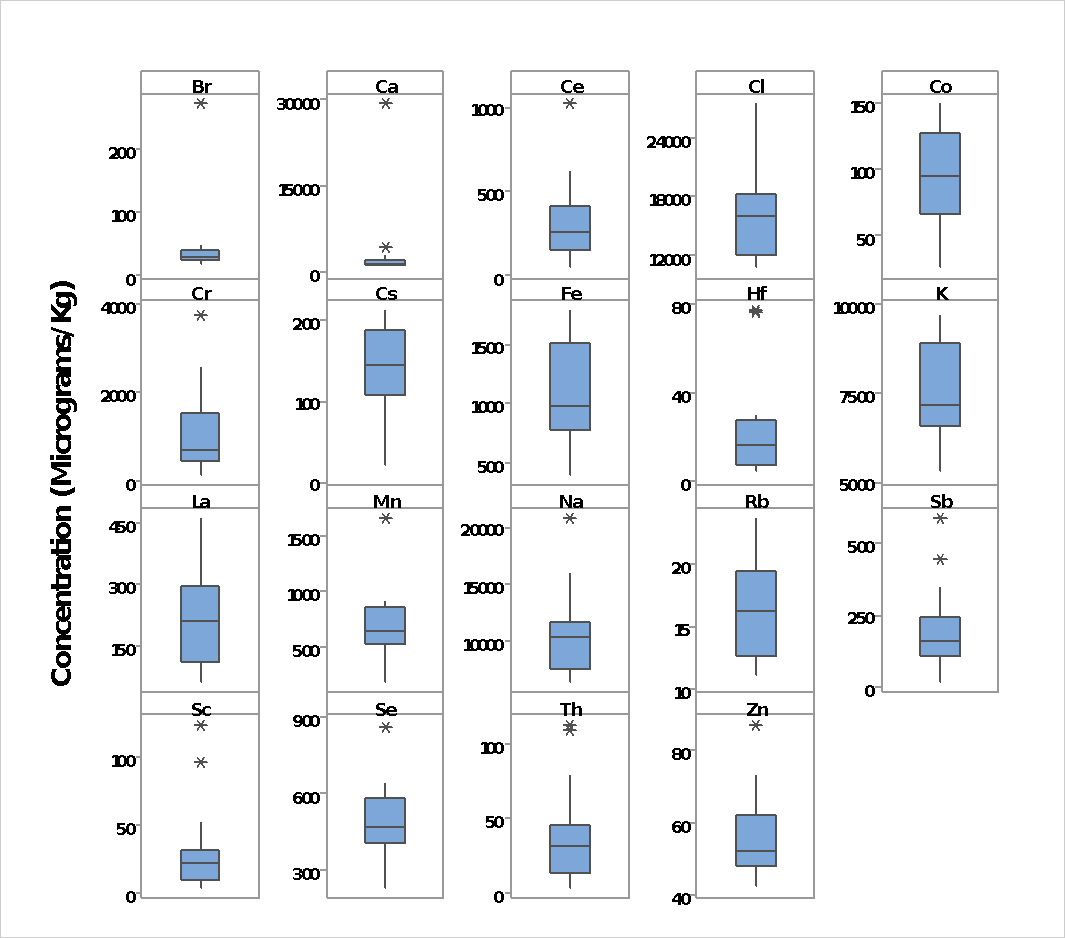


*Figure S2: Box-plots of chemical elements concentrations in the lung (µg / kg)*

Supplement: Supplementary file 1 — Supplementary Information 1. [file 41598_2022_15048_MOESM1_ESM.docx]

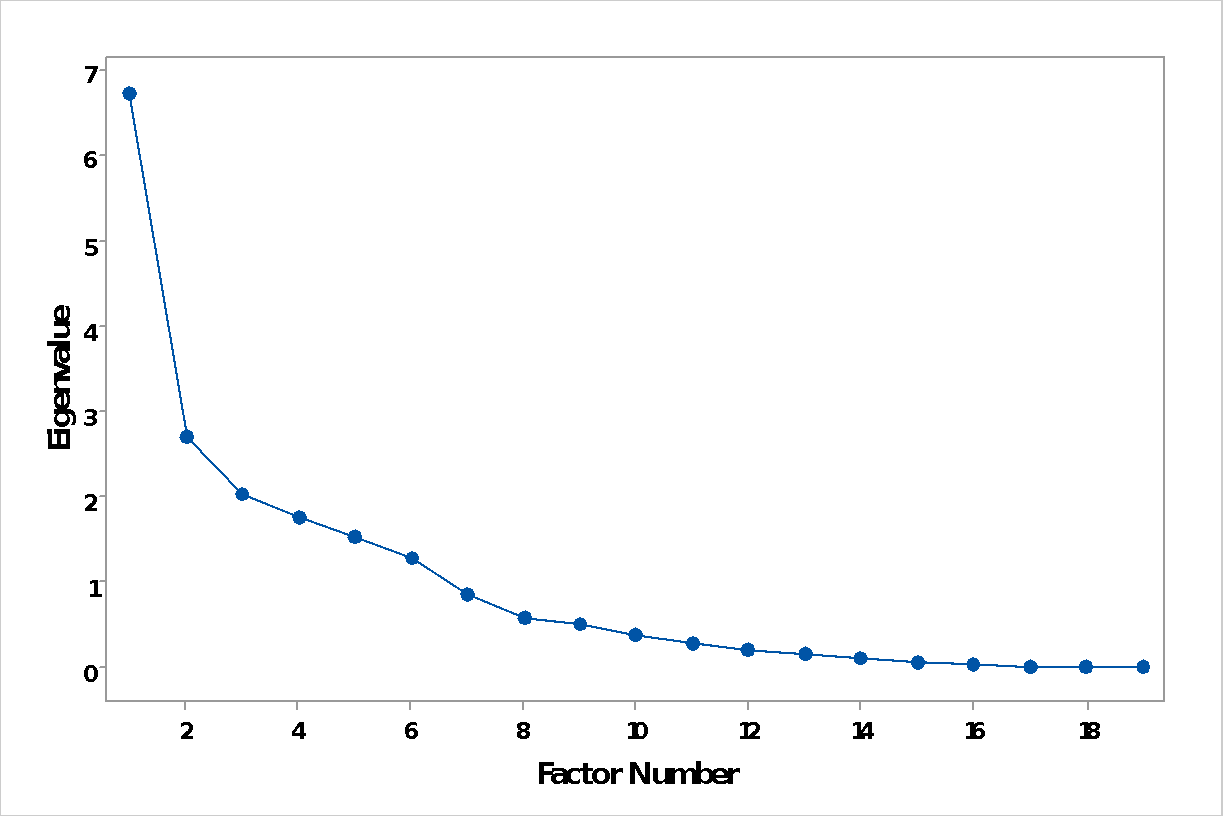


Figure S3- Scree plot obtained in the Factor analysis

Supplement: Supplementary file 3 — Supplementary Information 3. [file 41598_2022_15048_MOESM3_ESM.docx]
