# Supplementary material for: Accumulation of trace element content in the lungs of Sao Paulo city residents and its correlation to lifetime exposure to air pollution
Source: Sci Rep. 2022 Jun 30;12:11083. doi: 10.1038/s41598-022-15048-2 (PMC9247064; doi:10.1038/s41598-022-15048-2)
Supplement: Supplementary file 2 — Supplementary Information 2. [file 41598_2022_15048_MOESM2_ESM.docx]

Supplementary Figures

Figure S1 – Box-plot of Polonium 210 * over study period
