# Supplementary material for: Accumulation of trace element content in the lungs of Sao Paulo city residents and its correlation to lifetime exposure to air pollution
Source: Sci Rep. 2022 Jun 30;12:11083. doi: 10.1038/s41598-022-15048-2 (PMC9247064; doi:10.1038/s41598-022-15048-2)
Supplement: Supplementary file 4 — Supplementary Information 4. [file 41598_2022_15048_MOESM4_ESM.docx]

*Table S1:* Spearman correlation coefficients between the concentrations of the elements.

| **Spearman correlation** | Mean | Lung_Co | Lung_Th | Lung_Sb | Lung_Sc | Lung_Hf | Lung_Cr | Lung_K | Lung_Rb | Lung_Mn | Lung_Cl | Lung_Na | Lung_Cs | Lung_Br | Lung_Ce | Lung_La | Lung_Se | Lung_Ca | Lung_Zn | Lung_Fe | Po210  Lung |
| --- | --- | --- | --- | --- | --- | --- | --- | --- | --- | --- | --- | --- | --- | --- | --- | --- | --- | --- | --- | --- | --- |
| Mean | **1** |  |  |  |  |  |  |  |  |  |  |  |  |  |  |  |  |  |  |  |  |
| Lung_Co | **0,491*** | 1 |  |  |  |  |  |  |  |  |  |  |  |  |  |  |  |  |  |  |  |
| Lung_Th | **0,588**** | 0,877 | 1 |  |  |  |  |  |  |  |  |  |  |  |  |  |  |  |  |  |  |
| Lung_Sb | **0,518*** | 0,528 | 0,684 | 1 |  |  |  |  |  |  |  |  |  |  |  |  |  |  |  |  |  |
| Lung_Sc | **0,553*** | 0,771 | 0,887 | 0,63 | 1 |  |  |  |  |  |  |  |  |  |  |  |  |  |  |  |  |
| Lung_Hf | **0,587*** | 0,624 | 0,777 | 0,695 | 0,8 | 1 |  |  |  |  |  |  |  |  |  |  |  |  |  |  |  |
| Lung_Cr | 0,303 | 0,529 | 0,569 | 0,7 | 0,474 | 0,578 | 1 |  |  |  |  |  |  |  |  |  |  |  |  |  |  |
| Lung_K | -0,144 | -0,22 | -0,334 | -0,564 | -0,371 | -0,333 | -0,231 | 1 |  |  |  |  |  |  |  |  |  |  |  |  |  |
| Lung_Rb | -0,159 | -0,513 | -0,498 | -0,546 | -0,298 | -0,28 | -0,327 | 0,274 | 1 |  |  |  |  |  |  |  |  |  |  |  |  |
| Lung_Mn | -0,062 | 0,188 | 0,389 | 0,514 | 0,412 | 0,624 | 0,369 | -0,389 | -0,365 | 1 |  |  |  |  |  |  |  |  |  |  |  |
| Lung_Cl | -0,371 | -0,397 | -0,265 | 0,128 | -0,302 | -0,519 | -0,038 | -0,164 | -0,377 | 0,152 | 1 |  |  |  |  |  |  |  |  |  |  |
| Lung_Na | -0,238 | -0,532 | -0,365 | -0,041 | -0,465 | -0,564 | -0,105 | 0,042 | -0,239 | -0,03 | 0,768 | 1 |  |  |  |  |  |  |  |  |  |
| Lung_Cs | 0,241 | 0,229 | 0,262 | 0,364 | 0,585 | 0,49 | 0,335 | -0,232 | 0,105 | 0,362 | -0,217 | -0,34 | 1 |  |  |  |  |  |  |  |  |
| Lung_Br | 0,082 | 0,223 | 0,504 | 0,296 | 0,496 | 0,399 | -0,046 | -0,239 | -0,206 | 0,356 | 0,072 | -0,132 | 0,065 | 1 |  |  |  |  |  |  |  |
| Lung_Ce | 0,204 | 0,304 | 0,367 | 0,037 | 0,407 | 0,23 | 0,226 | 0,33 | -0,098 | 0,153 | -0,189 | -0,386 | 0,251 | 0,281 | 1 |  |  |  |  |  |  |
| Lung_La | 0,265 | 0,268 | 0,549 | 0,287 | 0,674 | 0,383 | 0,259 | -0,11 | 0,006 | 0,349 | -0,068 | -0,335 | 0,362 | 0,623 | 0,691 | 1 |  |  |  |  |  |
| Lung_Se | -0,147 | 0,041 | -0,159 | 0,01 | -0,224 | -0,158 | -0,118 | -0,083 | -0,173 | -0,13 | -0,08 | 0,074 | -0,174 | -0,423 | -0,479 | -0,469 | 1 |  |  |  |  |
| Lung_Ca | -0,165 | -0,165 | -0,086 | 0,22 | -0,152 | -0,121 | -0,009 | -0,051 | -0,337 | 0,008 | 0,253 | 0,379 | 0,09 | 0,026 | -0,021 | -0,25 | 0,112 | 1 |  |  |  |
| Lung_Zn | -0,035 | 0,032 | 0,072 | 0,168 | 0,132 | 0,051 | -0,092 | -0,045 | -0,316 | 0,435 | 0,071 | 0,033 | 0,311 | 0,02 | 0,219 | 0,006 | 0,111 | 0,583 | 1 |  |  |
| Lung_Fe | 0,103 | 0,368 | 0,37 | -0,048 | 0,334 | 0,23 | -0,076 | 0,035 | 0,095 | 0,054 | -0,328 | -0,305 | -0,147 | 0,34 | 0,075 | 0,168 | -0,144 | -0,423 | -0,191 | 1 |  |
| Po210  Lung | -0,032 | 0,14 | 0,343 | 0,386 | 0,254 | 0,282 | **0,599**** | -0,065 | -0,087 | **0,555**** | 0,101 | -0,117 | 0,122 | 0,108 | 0,346 | **0,502*** | -0,334 | -0,174 | 0,038 | -0,18 | 1 |
